# Thicker Inner Nuclear Layer as a Predictor of Glaucoma Progression and the Impact of Intraocular Pressure Fluctuation

**DOI:** 10.3390/jcm13082312

**Published:** 2024-04-17

**Authors:** Kyoung In Jung, Hee Kyung Ryu, Si Eun Oh, Hee Jong Shin, Chan Kee Park

**Affiliations:** Department of Ophthalmology, Seoul St. Mary’s Hospital, College of Medicine, The Catholic University of Korea, Seoul 06591, Republic of Korea; ezilean@hanmail.net (K.I.J.); heekyung130@naver.com (H.K.R.); sieeun5151@naver.com (S.E.O.); shinhj01@naver.com (H.J.S.)

**Keywords:** glaucoma, progression, inner nuclear layer thickness, microcystic macular changes

## Abstract

**Background**: Thickening of the inner nuclear layer (INL) or microcystic macular changes has been reported to be implicated in glaucoma patients, but their potential impact on disease progression remains unclear. We investigated the relationship between baseline microcystic macular edema in the INL or INL thickness and subsequent visual field (VF) progression in glaucoma patients. **Methods**: This retrospective observational study included primary open-angle glaucoma with follow-up exceeding 3 years. We identified macular cystic changes through Spectralis optical coherence tomography and measured the INL thickness using automated segmentation. Glaucoma progression was determined using the Guided Progression Analysis program of the Humphrey filed analyzer, calculating the mean deviation (MD) changes (dB/year). **Results**: Microcystic macular changes were observed in 12 (7.5%) of 162 patients. Patients with microcystic macular change had thicker INL thickness than those without it (*p* = 0.010). Progressors had a higher probability of having microcystic macular changes and a thicker average INL thickness than nonprogressors (*p* = 0.003, *p* = 0.019). Thicker INL thickness was associated with faster VF progression based on MD slope (dB/year) in the multivariate regression analysis (*p* = 0.045). Additionally, greater intraocular pressure (IOP) fluctuation was found to be associated with both a thicker INL and the presence of microcystic changes in the multivariate regression analysis (*p* = 0.003, 0.028). **Conclusions**: Increased macular INL thickness indicative of INL changes was linked to subsequent VF progression in glaucoma patients. These findings suggest that retinal inner nuclear change could serve as an indicator of progressive glaucoma.

## 1. Introduction

Glaucomatous optic neuropathy is characterized by selective loss of retinal ganglion cells (RGCs). In glaucoma, the degeneration of the axons, cell bodies, and dendrites of RGCs was observed mainly from the retinal nerve fiber layer (RNFL) to the inner plexiform layer (IPL), although outer retinal layer changes have been reported in a few studies [1,2].

A growing body of evidence has reported that microcystic macular changes were found in the inner nuclear layer (INL) in up to 10% of patients with several types of optic nerve diseases, including glaucoma [3]. RGCs synapse or connect to bipolar cells, amacrine cells, and Müller cells in the IPL, and their cell bodies were located in the INL. Retrograde transcellular degeneration of cells in the INL secondary to RGC loss, vitreoretinal traction, and Müller cell dysfunction has been suggested as the mechanism of microcystic macular changes detected in optic nerve degeneration [3,4,5,6]. The precise mechanism of experiencing microcystic macular changes in the INL has not been elucidated clearly.

It has been reported that microcystic macular changes were associated with a thinner RNFL or ganglion cell layer (GCL) or more severe visual field (VF) loss in optic neuropathies [3,4,6,7]. Our group previously found that a thicker INL correlates with more severe VF damage in patients with glaucoma [8]. Regarding the progression of glaucoma, one study reported that the presence of microcystic macular changes is associated with faster VF progression [9]. Another study found that the rate of global MD or VF index did not significantly differ according to the presence of microcystic macular changes, although superior central VF damage deteriorated rapidly in glaucoma patients with microcystic macular changes [7].

Microcystic macular changes were found to be associated with INL thickening in optic neuropathies of variable etiologies [3,4,5,6,9]. The presence of microcysts itself in the INL might contribute to an increased thickness of the INL [4,9]. In patients with multiple sclerosis, the INL was thicker in patients with relapsing–remitting multiple sclerosis than in healthy control subjects, even when microcystic macular changes had not been identified yet [5]. This finding suggests that INL thickening might precede the development of microcystic macular edema [5]. Having microcystic changes or not is a dichotomous classification partially dependent on optical coherence tomography (OCT) resolution. Quantitative assessment of the effects of INL thickness on glaucomatous VF progression might yield a clue about the pathogenesis of microcystic macular changes in the INL, and the existence of a correlation between the INL thickness and VF progression could reveal a biomarker for glaucoma progression. No study has evaluated the relationship between INL thickness and VF progression in patients with glaucoma.

In this study, we investigated the association between the presence of INL microcystic change or INL thickness and subsequent VF progression. Additionally, we tried to uncover the factors related to the presence of microcystic change or INL thickness in patients with glaucoma.

## 2. Materials and Methods

The institutional review board of the Catholic University of Korea, Seoul, Republic of Korea, approved this retrospective observational study and waived the need for written informed consent because of its retrospective design. This study followed the tenets of the Declaration of Helsinki. Among patients who visited a glaucoma clinic of Seoul St. Mary’s Hospital (Seoul, Republic of Korea) between January and March 2015, patients with a diagnosis of primary open-angle glaucoma who were imaged with the Spectralis spectral-domain OCT (SD-OCT) system (Heidelberg Engineering, Heidelberg, Germany) and met the inclusion criteria were enrolled into this study. The study inclusion criteria were the presence of an open angle under gonioscopic examination, axial length < 28 mm, and >3 years of follow-up. Exclusion criteria were corneal disease affecting vision; a history of or current uveitis, diabetic retinopathy, or other vitreoretinal diseases (e.g., retinal vein obstruction, central serous chorioretinopathy, age-related macular degeneration, retinal detachment); brain disease that could impact visual function; and a history of glaucoma or posterior segment surgery. Abnormal findings in the macular region, such as retinoschisis or cystoid macular edema after intraocular surgery, were also selected as exclusion criteria. Patients with an epiretinal membrane (ERM) were not excluded. Patients with a kind of optic nerve degeneration other than glaucoma, such as hereditary, inflammatory, or ischemic optic neuropathy, or optic disc drusen, were excluded.

All patients underwent a complete ophthalmic examination, including IOP measurement by Goldmann applanation tonometry, central corneal thickness measurement, axial length measurement, stereoscopic optic disc photography, and red-free photography. During their first visit to our clinic, each patient underwent blood pressure measurement in the sitting position using a standard automated blood pressure cuff after a 5 min rest. Eyes with a glaucomatous optic disc, such as rim loss, notching, and RNFL defects corresponding to VF loss in standard automated perimetry 24-2, were diagnosed as having glaucoma.

Long-term IOP fluctuation was determined as the standard deviation (SD) of all IOP values during visits. The high IOP fluctuation group and the low IOP fluctuation group were differentiated based on the median value of the IOP SD values of all patients.

### 2.1. OCT and Automated Segmentation of the Retina

**Figure 1:**
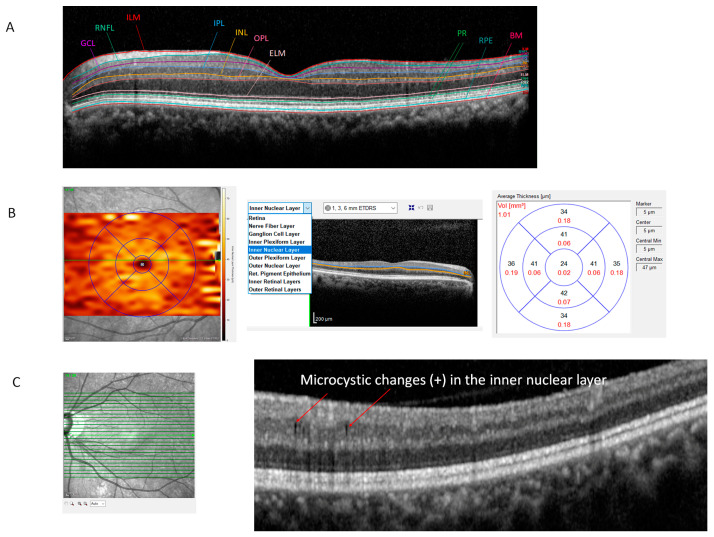
Segmentation of the retinal layers. (**A**) Segmentation was carried out automatically by a segmentation application (Segmentation Technology; Heidelberg Engineering). The automated segmentation program shows 11 different retinal demarcation lines: the inner limiting membrane, the boundaries between the RNFL and the ganglion cell layer (GCL); between the GCL and the inner plexiform layer (IPL); between the IPL and the inner nuclear layer (INL); between the INL and the outer plexiform layer; between the outer plexiform layer and the outer nuclear layer; and between the external limiting membrane, two photoreceptor layers (PR1/2), the RPE, and Bruch’s membrane. (**B**) After automatic segmentation of the retinal layers, thickness measurements were displayed for every segmented retinal layer inside of the nine 1, 3, and 6 mm grid sectors defined by the Early Treatment Diabetic Retinopathy Study (ETDRS) grid on the thickness map. (**C**) Microcystoid macular change (arrow) was defined by the presence of multiple, small hyporeflective round or elliptical cystoid spaces without a cyst wall observed in the INL and not confluent with cystoid spaces in other retinal layers.

All subjects were examined with the Spectralis SD-OCT system (Heidelberg Engineering, Heidelberg, Germany) using the fast macular cube protocol. Real-time eye-tracking software yielded perifoveal volumetric retinal scans consisting of 25 single horizontal axial scans (scanning area, 666 mm^2^ centered at the fovea). Segmentation of the retinal layers was carried out automatically by a segmentation application (Segmentation Technology; Heidelberg Engineering). The automated segmentation program shows 11 different retinal demarcation lines: the inner limiting membrane, the boundaries between the RNFL and the ganglion cell layer (GCL); between the GCL and the inner plexiform layer (IPL); between the IPL and the INL; between the INL and the outer plexiform layer; between the outer plexiform layer and the outer nuclear layer; the external limiting membrane, two photoreceptor layers (PR1/2), the RPE, and Bruch’s membrane (Figure 1A). After automatic segmentation of the retinal layers, thickness measurements were displayed for every segmented retinal layer inside of the nine 1, 3, and 6 mm grid sectors defined by the Early Treatment Diabetic Retinopathy Study (ETDRS) grid on the thickness map (Figure 1B).

The segmentation process revealed each retinal layer thickness, including the overall retinal thickness and those of the RNFL, GCL, IPL, INL, outer plexiform layer, outer nuclear layer, outer limiting membrane, photoreceptor layers, retinal pigment epithelium, inner retinal layer, and outer retinal layer. The average total retinal, RNFL, GCL, IPL, and INL thickness values in nine sectors and the total volume were calculated and adopted in this study. The total volume of each retinal layer within the ETDRS circle was displayed on the thickness map.

The occurrence of microcystoid macular change was defined by the presence of multiple, small hyporeflective round or elliptical cystoid spaces without a cyst wall detected in the INL and not confluent with cystoid spaces in other retinal layers (Figure 1C) [10]. To avoid a false-positive result in the decision about the presence of microcysts, microcystoid macular change was determined to be present when microcysts were observed on more than two serial scans and when two observers (C.K.P. and K.I.J.) agreed on the presence of microcystoic macular changes.

### 2.2. VF Testing

All patients underwent an standard automated perimetry 24-2 test with a Humphrey field analyzer (Carl Zeiss Meditec, Doublin, CA, USA) using the Swedish interactive threshold algorithm standard strategy. Glaucomatous VF defects were defined as clusters of at least three points having sensitivities of <5% of the normal population on the pattern deviation plot. A single abnormal point should have a sensitivity of <1% of the normal population. The mean deviation (MD) and pattern standard deviation were analyzed. Reliable VFs were considered those with <15% fixation losses, false positives, or false negatives. A VF test performed from January to March 2015 was designated as the baseline VF test because segmentation of the retinal layers by the Spectralis SD-OCT system was available from January 2015 in our clinic. The baseline VF test and Spectralis SD-OCT exam were performed on the same day.

Progression of glaucoma was determined when “likely progression” alerts were shown by the Guided Progression Analysis program of the Humphrey field analyzer. The Guided Progression Analysis program is based on the Early Manifest Glaucoma Trial (EMGT) criteria (event-based analysis), which indicate progression when three or more points show a significant deterioration (at the *p* < 0.05 level) in at least three consecutive examinations compared to two baseline pattern deviation VFs [11]. Calculation of VF change rates was performed using MD change (dB) per year during follow-up periods.

### 2.3. Statistical Analysis

SPSS software (version 24.0; IBM Corporation, Armonk, NY, USA) was applied for statistical tests. Differences among the groups were assessed by independent *t*-test or the chi-squared test. Test–retest variability was determined using the intraclass correlation coefficient (ICC), with scores of ≥0.75, 0.40–0.75, and ≤0.40 points indicating excellent, moderate, and poor variability, respectively [12]. Correlations between variables were evaluated with Pearson correlation coefficients. Multivariate regression analysis was performed to compute the factors associated with the MD slope (MD change per year). Multiple logistic regression analysis was run to determine the factors associated with VF progression based on EMGT criteria. Factors with a difference of *p* < 0.05 between two groups were added into the multivariate regression analysis. *p* < 0.05 was considered to represent statistical significance.

## 3. Results

The mean age of the 162 patients enrolled in this study was 54.2 ± 13.6 years, and the baseline MD of the VF 24-2 test result was −4.0 ± 4.4 dB (Table 1). Among glaucoma patients, 139 patients (85.8%) were diagnosed with normal-tension glaucoma, with the remaining 23 patients (14.2%) diagnosed with primary open-angle glaucoma.

Each inner retinal layer thickness displayed excellent reproducibility for total retinal, RNFL, GCL, IPL, and INL thickness and volume measurements (ICC = 0.778–0.960; Table 2).

The baseline retinal thickness, RNFL thickness, GCL thickness, and IPL thickness each had a positive correlation with the baseline VF MD (r = 0.287–0.412, all *p* < 0.05; Table 3). Conversely, the baseline INL thickness had a negative correlation with the baseline VF MD (r = −0.166, *p* = 0.035).

Microcystic macular changes were observed in 12 (7.5%) of the 162 study participants. No patients showed microcystic macular changes on the fovea, and the mean distance from the foveal center to the nearest microcyst was 900 μm (453–1479 μm). Microcystic INL changes were found mostly in the inferior hemisphere (10 eyes, 93.3%), with one eye (8.3%) having changes in the superior hemisphere and one eye (8.3%) displaying changes in both hemispheres. Based on the foveal center, microcystic changes were predominantly found in the temporal area (7 eyes, 58.3%), being less common on the nasal side (2 eyes, 16.7%) or both sides (3 eyes, 25.0%). A correspondence between the location of microcystic changes (superior/inferior/both) and predominant VF defect location (superior/inferior/both) was observed in eight eyes (66.7%).

Patients with microcystic macular changes displayed greater proportions of having diabetes or a higher IOP SD, being in the high IOP fluctuation group based on the IOP median, having ERM, and having a lower baseline VF MD or a higher baseline PSD than those without microcystic macular changes (*p* = 0.004, *p* = 0.032, *p* = 0.005, *p* = 0.003, *p* = 0.003, and *p* = 0.008; Table 4).

Patients with microcystic macular changes had thicker average INLs and greater INL volumes than those without microcystic macular changes (*p* = 0.010 and *p* < 0.001, respectively; Table 5).

Of 162 total patients, 33 (20.4%) patients demonstrated VF progression (Table 6). The proportion of having cold hands was higher in the progressors (30.3%) compared to the nonprogressors (14.0%, *p* = 0.029).

Patients showing glaucoma progression had a thicker average INL and greater INL volume than those without glaucoma progression (*p* = 0.019 and *p* = 0.015; Table 7). The average INL thickness displayed a negative correlation with the MD slope (dB/year) (r = −0.244, *p* = 0.002). The presence of both microcystic macular changes and ERM was found more frequently in the progression group (21.2% and 12.1%) than in the nonprogression group (3.9% and 0.8%) (*p* = 0.003 and *p* = 0.006).

The univariate regression analysis (trend-based analysis) to determine the factors associated with MD slope (dB/year) revealed that patients showing microcystic macular changes, ERM, or a thicker INL were likely to have VF deterioration based on the MD slope (*p* = 0.016, *p* = 0.011, and *p* = 0.002, respectively; Table 8). However, only a thicker INL remained a relevant factor for VF progression in the multivariate regression analysis (*p* = 0.045). In the logistic regression analysis based on EMGT criteria (event-based analysis), having cold hands, microcystic macular changes, ERM, or a thicker INL, respectively, was associated with VF deterioration (*p* = 0.031, *p* = 0.002, *p* = 0.012, and *p* = 0.027, respectively). In the multiple logistic regression analysis, no factors showed a significant correlation with glaucoma progression, although the presence of microcystic macular changes was marginally associated with VF deterioration (*p* = 0.051).

**Figure 2:**
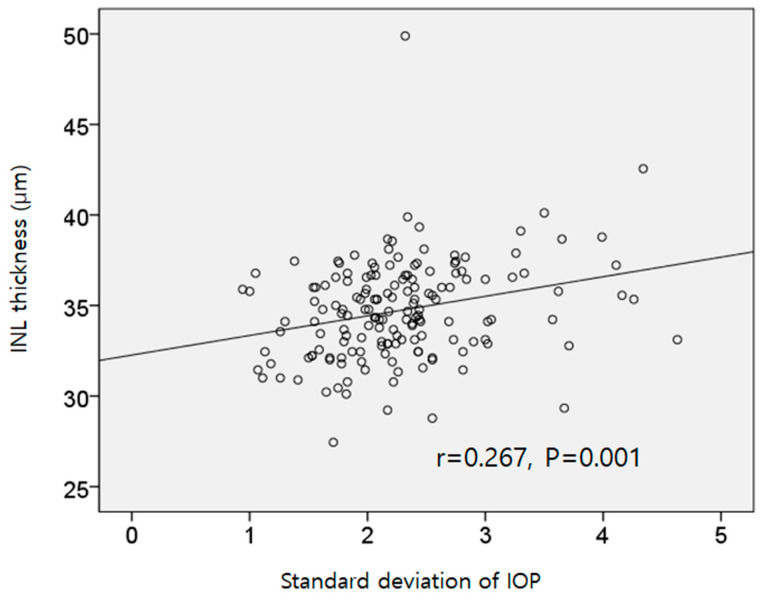
The relationship between intraocular pressure (IOP) fluctuations and the macular inner nuclear layer (INL) thickness. A higher standard deviation of IOP was associated with a thicker INL (r = 0.267, *p* = 0.001).

Regarding the factors related to average INL thickness, a higher IOP SD and the presence of ERM or microcystic macular changes were associated with a thicker INL in the multivariate regression analysis (*p* = 0.003, *p* < 0.001, and *p* = 0.004; Table 9, Figure 2). In the subgroup analysis of patients without microcystic macular changes (*n* = 150), a shorter axial length and higher IOP SD were significantly related to a thicker INL in the multivariate regression analysis (*p* = 0.026 and *p* = 0.001, respectively).

Multiple logistic regression analysis revealed that having diabetes, ERM, or a higher IOP fluctuation correlates with the presence of microcystic macular changes in glaucoma patients (*p* = 0.021, *p* = 0.027, and *p* = 0.028, respectively; Table 10).

**Figure 3:**
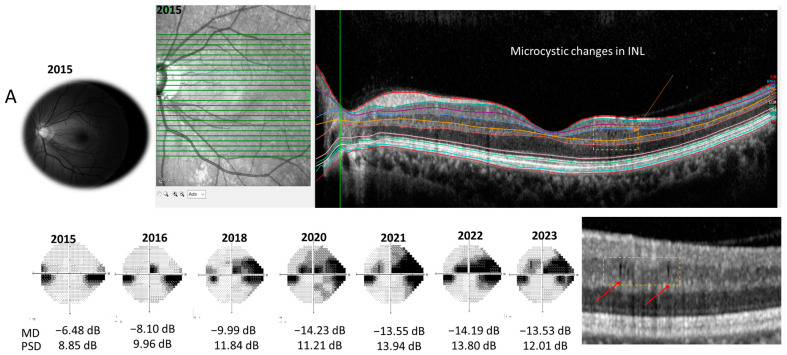

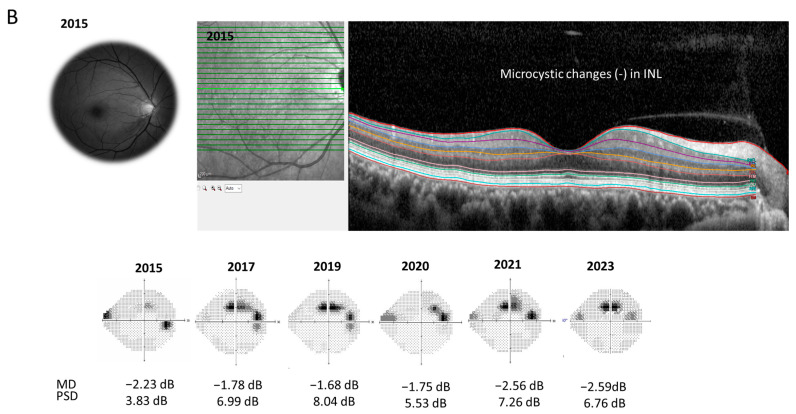
A representative case. (**A**) A 36-year-old female patient showed macular microcystic changes (arrow) in the inner nuclear layer (INL) and moderate glaucomatous visual field (VF) defects in 2015. The average INL thickness was 37.3 μm. The patient showed VF progression from 2015 to 2023. (**B**) A 51-year-old female patient had early glaucomatous VF defects and did not display microcytic macular changes in the INL. The average INL thickness was 32.1 μm, which is relatively thin. Her VF defects did not progress significantly.

Representative cases are shown in Figure 3. A 36-year-old female patient with normal-tension glaucoma showed a moderate glaucomatous VF defect (MD = −6.48 dB, PSD = 8.85 dB). Progressive VF changes were observed along with microcystic macular changes in the INL of her left eye (Figure 3A). A 51-year-old female patient had early glaucomatous VF defects without microcytic macular changes in the INL (Figure 3B). Her VF defects did not progress rapidly. The average INL thickness was 37.3 μm in the first case (A) and 32.1 μm in the second case (B).

## 4. Discussion

In this study, glaucoma patients who showed VF progression had a higher probability of exhibiting microcystic macular changes and a greater INL thickness compared to nonprogressors. A thicker INL remained to be associated with faster VF progression in multivariate analysis. Furthermore, greater IOP fluctuation was found to be related to both a thicker INL and the presence of microcystic changes.

Patients with thicker INLs displayed more severe glaucomatous VF loss. Macular INL thickness was negatively related to baseline VF MD, whereas macular RNFL, GCL, and IPL thickness were positively associated with VF MD. This corresponds with our previous study showing the negative correlation between INL thickness and the corresponding paracentral VF sensitivities [8]. In patients with multiple sclerosis, an increased INL thickness is associated with more severe disease activity [5].

In the present study, microcystic macular changes were observed in 12 patients (7.5%). The prevalence of microcystic macula was about 2.8–8%, which can differ depending on OCT resolution and whether patients with ERM were excluded or not [3,6,7,9]. Patients with microcystic macular changes in the INL had more severe VF damage based on VF MD and PSD. The correlation between the presence of microcystic macular changes and more severe glaucomatous damage corresponds with findings of prior studies enrolling glaucoma patients [3,6,7,9]. The reason why microcystic changes in the INL or an increased INL thickness are found in diseases with optic nerve degeneration has not been elucidated definitively. Microcystic macular changes have not been linked to vascular leakage and do not involve the fovea, unlike the macular edema attributable to diabetic retinopathy or retinal vein occlusion [3,4]. In this study, there was no significant difference in best-corrected visual acuity between patients with and without microcystic macular changes (*p* = 0.589). Retinal vascular leakage can be excluded as the cause of microcystic changes in glaucoma patients because fluorescein angiography did not reveal dye leakage [3,13]. Several mechanisms have been suggested, such as vitreomacular traction, trans-synaptic retrograde degeneration of cells in the INL, and degeneration of Müller cells [3,4,5,6].

The presence of microcystic macular changes in the INL was marginally associated with VF deterioration based on event-based analysis (*p* = 0.051). The relationship between microcystic macular changes and VF progression in glaucoma is controversial. Hasegawa et al. reported that the VF MD slope was significantly worse in eyes with microcytic lesions (*p* = 0.013) [9]. Mahmoudinezhad et al. found that the presence of microcystic macular changes was not associated with MD and VFI decay rates, even though it is related to quicker VF progression in the superior central area [7].

In the multivariate analysis, an increased INL thickness was found to be independently associated with faster VF progression in glaucoma patients regardless of the presence of microcystic macular changes or ERM, according to the trend-based analysis (*p* = 0.045). This is noteworthy, even though a thicker INL was associated with the presence of microcystic changes in the univariate analysis. In patients with multiple sclerosis, increased baseline INL thickness predicts the occurrence of relapse, new brain magnetic resonance imaging T2 lesions, and disability progression, whereas the presence of microcystic macular edema is not associated with radiological disease activity or disability progression, consistent with our study [5]. No study to date has investigated the relationship between INL thickness and glaucoma progression, and the reason why glaucoma progression is associated with increased INL thickness rather than the presence of microcystic macular changes is not clear now. We speculate that an increased INL thickness might develop earlier than microcysts, or OCT resolution cannot detect the presence of very tiny microcysts in the INL when the INL thickness has already increased. Other mechanisms might exist in INL thickening, which is different from the formation of microcysts.

Regarding the factors related to INL thickness, higher IOP fluctuation was associated with a thicker INL in glaucoma patients with or without microcystic macular changes in the INL. Higher IOP fluctuations have been found to be associated with glaucoma because IOP fluctuates more in glaucoma patients than in healthy control subjects [14,15,16,17]. Controversies have existed about the effects of IOP fluctuation on glaucoma progression, even though the Advanced Glaucoma Intervention Study supported the relationship between greater IOP fluctuation and VF progression [18,19,20]. In the present study, IOP fluctuation itself was not directly related to the progression of glaucoma but showed relevance to increased INL thickness, which was a determinant of predicting more rapid VF progression. Higher IOP fluctuation could induce a kind of reactive gliosis in the retina or optic nerve head [21,22]. In a rodent glaucoma model with chronic ocular hypertension, hypertrophy of Müller cells was found from the inner limiting membrane to the outer nuclear layer, detected by increased expression of glial fibrillary acidic protein. In a glaucoma model, the dysfunction of Müller cells can be suspicious because they do not sustain increased glutamate/aspartate transporter activity as excitotoxicity is aggravated [23]. The cell bodies of Müller cells are located in the INL, and they play a role in fluid absorption of the inner retina through aquaporin channels or potassium channels [3,4,24]. We speculated that greater IOP fluctuation in glaucoma may lead to increased INL thickness, possibly by reactive gliosis or Müller cell dysfunction. Whether glial reactivation is protective or detrimental to RGCs remains unconfirmed. In a subgroup analysis of glaucoma patients without microcystic macular changes, patients with longer axial lengths had a higher probability of reduced INL thickness. Several studies have demonstrated that the INL is thinner in highly myopic eyes compared to emmetropic eyes, possibly because the INL could be made thinner by stretching [25,26].

The factors in the univariate logistic regression analysis that correlated with the presence of microcystic changes in the INL were diabetes, ERM, worse baseline MD, and higher IOP fluctuation. Among them, diabetes, ERM, and higher IOP fluctuation remained factors associated with the presence of microcystic changes in the multiple logistic regression analysis. Regarding diabetes, patients with diabetic retinopathy were excluded from this study, and microcystic changes in patients with diabetes did not involve the fovea. Previously, we reported that diabetic rats showed increased IOP fluctuations, RGC apoptosis, gliosis, and neuroinflammation [27]. Therefore, subclinical inflammation or IOP fluctuations associated with diabetes might affect the formation of microcystic changes in the INL, even though further studies are acquired to elucidate the precise mechanisms. Govetto et al. found that the likelihood of developing microcystic macular changes in the INL was greater in patients with advanced ERM stages and glaucoma, consistent with our study [24]. Mahmoudinezhad et al. reported no association between ERM and the presence of microcystic macular changes [7]. Given these findings and our results, the presence of ERM might help to decrease the threshold for the formation of microcystic macular changes, and other mechanisms may also play a role in the formation of microcystic changes in glaucoma patients. Being in the higher IOP fluctuation group (established based on the median SD of long-term IOP measurements) was associated with the presence of microcystic macular changes. Greater IOP fluctuation might induce a kind of reactive gliosis in the retina and could affect the formation of microcystic changes, as we assumed about the relationship between the IOP fluctuation and INL thickness, although this is just a speculation [21,22]. Further studies need to confirm the specific mechanism in the relationship between IOP fluctuation and microcystic changes or INL thickness. We found no relationship between age and the presence of microcystic changes, in accordance with the study by Hasegawa et al. [9], whereas another study reported that younger age correlates with microcystic changes [7]. Previous studies did not include systemic diseases such as diabetes or IOP fluctuations as variables when investigating factors related to the presence of microcystic changes in the INL [7,9].

To decrease bias associated with segmentation errors in automated layer segmentation, we confirmed the segmentation of all OCT scans and excluded the cases with artifacts or misalignment. In this study, we checked the variability of each retinal layer thickness, and each inner retinal layer thickness displayed excellent reproducibility.

## 5. Conclusions

A thicker INL was associated with subsequent VF progression in glaucoma patients. We found that higher IOP fluctuation contributes to both increased INL thickness and the formation of microcystic changes in glaucoma. Measurements of macular INL thickness or observation of microcystic changes might be helpful in predicting the future progression of glaucoma, especially in glaucoma patients with greater IOP fluctuation. Further prospective studies are necessary to establish the mechanisms behind increased INL thickness or the formation of microcysts and reveal longitudinal INL changes in glaucoma patients.

## Figures and Tables

**Table 1:** Demographics of total patients with glaucoma.

	Eyes with Glaucoma (*n* = 162)
Age (years)	54.2 ± 13.6
Male/Female	78/84
Diabetes (%)	25 (15.4%)
Systemic hypertension (%)	44 (27.2%)
Migraine (%)	30 (18.5%)
Cold hand (%)	28 (17.3%)
Cardiovascular disease (%)	22 (13.6%)
Thyroid disease	18 (11.1%)
Systolic blood pressure (mmHg)	126.4 ± 15.0
Diastolic blood pressure (mmHg)	79.1 ± 8.4
Body mass index (kg/m^2^)	23.3 ± 3.0
Central corneal thickness (µm)	536.4 ± 35.7
Axial length (mm)	24.8 ± 1.5
Spherical equivalent (diopter)	−2.5 ± 3.5
Mean IOP (mmHg)	14.7 ± 2.4
SD of IOP (mmHg)	2.3 ± 0.7
Presence of disc hemorrhage (%)	21 (13.3%)
Presence of microcystic macular changes	12 (7.4%)
Baseline MD of VF 24-2 (dB)	−4.0 ± 4.4
Baseline PSD of VF 24-2 (dB)	4.7 ± 4.0

IOP, intraocular pressure; MD, mean deviation; PSD, pattern standard deviation; SD, standard deviation.

**Table 2:** Reliability of each inner retinal layer thickness.

OCT Parameters		ICC	95% CI	*p* Value
Retina	Average (µm)	0.906	0.821–0.951	**<0.001**
	Volume (mm^3^)	0.960	0.923–0.980	**<0.001**
RNFL	Average (µm)	0.778	0.604–0.882	**<0.001**
	Volume (mm^3^)	0.907	0.823–0.952	**<0.001**
GCL	Average (µm)	0.928	0.863–0.963	**<0.001**
	Volume (mm^3^)	0.951	0.905–0.975	**<0.001**
IPL	Average (µm)	0.950	0.903–0.974	**<0.001**
	Volume (mm^3^)	0.960	0.922–0.980	**<0.001**
INL	Average (µm)	0.884	0.782–0.940	**<0.001**
	Volume (mm^3^)	0.917	0.840–0.957	**<0.001**

GCL, ganglion cell layer; ICC, intraclass correlation coefficient; INL, inner nuclear layer; IPL, inner plexiform layer; OCT, optical coherence tomography; RNFL, retinal nerve fiber layer. The variables that were statistically significant in the Pearson correlation coefficients were indicated in bold.

**Table 3:** The association between each retinal layer thickness and initial VF mean deviation.

OCT Parameters		r	*p* Value
Retinal thickness	Average (µm)	0.287	**<0.001**
	Volume (mm^3^)	0.323	**<0.001**
RNFL thickness	Average (µm)	0.295	**<0.001**
	Volume (mm^3^)	0.384	**<0.001**
GCL thickness	Average (µm)	0.406	**<0.001**
	Volume (mm^3^)	0.412	**<0.001**
IPL thickness	Average (µm)	0.377	**<0.001**
	Volume (mm^3^)	0.350	**<0.001**
INL thickness	Average (µm)	−0.166	**0.035**
	Volume (mm^3^)	−0.144	0.068

GCL, ganglion cell layer; INL, inner nuclear layer; IPL, inner plexiform layer; OCT, optical coherence tomography; RNFL, retinal nerve fiber layer; VF, visual field. The variables that were statistically significant in the Pearson correlation coefficients were indicated in bold.

**Table 4:** Baseline characteristics of glaucoma patients according to the presence of microcystic macular changes.

	Microcystic Macular Changes (−) (*n* = 150)	Microcystic Macular Changes (+) (*n* = 12)	*p* Value
Age (years)	54.1 ± 13.3	56.1 ± 16.8	0.628
Male/Female	72/78	6/6	1.000
Diabetes (%)	19 (12.7%)	6 (50.0%)	**0.004**
Systemic hypertension (%)	40 (26.7%)	4 (33.3%)	0.736
Migraine (%)	30 (20%)	0 (0%)	0.125
Cold hand (%)	25 (16.7%)	3 (25.0%)	0.437
Cardiovascular disease (%)	19 (12.7%)	3 (25.0%)	0.211
Thyroid disease	18 (12.0%)	0 (0.0%)	0.364
Systolic blood pressure (mmHg)	125.9 ± 15.1	134.6 ± 7.8	0.207
Diastolic blood pressure (mmHg)	79.0 ± 8.6	82.2 ± 3.5	0.416
Body mass index (kg/m^2^)	23.4 ± 3.1	22.8 ± 1.1	0.615
Central corneal thickness (µm)	535.8 ± 36.3	543.9 ± 32.6	0.497
Axial length (mm)	24.9 ± 1.5	24.9 ± 1.5	0.919
BCVA (log MAR)	0.03 ± 0.05	0.03 ± 0.05	0.589
Spherical equivalent (diopter)	−2.5 ± 3.5	−3.8 ± 4.4	0.246
Mean IOP (mmHg)	14.7 ± 2.4	14.7 ± 2.0	0.928
SD of IOP (mmHg)	2.2 ± 0.7	2.7 ± 0.8	**0.032**
IOP fluctuation (Higher/lower)	70 (46.7%)/80 (53.3%)	11 (91.7%)/1 (8.3%)	**0.005**
Presence of disc hemorrhage (%)	19 (13.4%)	1 (8.3%)	1.000
Presence of ERM	2 (1.3%)	3 (25.0%)	**0.003**
Baseline MD of VF 24-2 (dB)	−3.7 ± 4.2	−7.6 ± 5.0	**0.003**
Baseline PSD of VF 24-2 (dB)	4.5 ± 3.9	7.8 ± 4.6	**0.008**

BCVA, best-corrected visual acuity; ERM, epiretinal membrane; IOP, intraocular pressure; MD, mean deviation; PSD, pattern standard deviation; SD, standard deviation; VF, visual field. Statistically significant differences between two groups (*p* < 0.05) by Student’s *t*-test for continuous variables or chi-squared test for categorical data are indicated in bold.

**Table 5:** Each retinal layer thickness according to the presence of microcystic macular changes.

OCT Parameters		Microcystic Macular Change (−) (*n* = 150)	Microcystic Macular Change (+) (*n* = 12)	*p* Value
Retina	Average (µm)	302.06 ± 15.03	307.24 ± 25.99	0.283
	Volume (mm^3^)	8.30 ± 0.43	8.39 ± 0.66	0.509
RNFL	Average (µm)	23.88 ± 4.17	25.13 ± 7.23	0.351
	Volume (mm^3^)	0.80 ± 0.16	0.79 ± 0.19	0.566
GCL	Average (µm)	34.96 ± 5.84	33.50 ± 8.41	0.426
	Volume (mm^3^)	0.95 ± 0.15	0.92 ± 0.22	0.516
IPL	Average (µm)	30.56 ± 3.75	30.63 ± 5.62	0.950
	Volume (mm^3^)	0.82 ± 0.10	0.83 ± 0.15	0.748
INL	Average (µm)	34.43 ± 2.38	38.29 ± 4.28	**0.010**
	Volume (mm^3^)	0.96 ± 0.06	1.06 ± 0.10	**<0.001**

GCL, ganglion cell layer; INL, inner nuclear layerIPL, inner plexiform layer; RNFL, retinal nerve fiber layer;. Statistically significant differences between two groups (*p* < 0.05) by Student’s *t*-test for continuous variables are indicated in bold.

**Table 6:** Baseline characteristics of glaucoma patients according to progression of visual field defects.

	Nonprogressors (*n* = 129)	Progressors (*n* = 33)	*p* Value
Age (years)	54.6 ± 13.0	53.0 ± 15.8	0.546
Male/Female	65/64	13/20	0.330
Diabetes (%)	21 (16.3%)	4 (12.1%)	0.787
Systemic hypertension (%)	36 (27.9%)	8 (24.2%)	0.827
Migraine (%)	23 (17.8%)	7 (21.2%)	0.624
Cold hand (%)	18 (14.0%)	10 (30.3%)	**0.029**
Cardiovascular disease (%)	20 (15.5%)	2 (6.1%)	0.253
Thyroid disease	15 (11.6%)	3 (9.1%)	1.000
Systolic blood pressure (mmHg)	127.2 ± 15.3	122.4 ± 12.1	0.279
Diastolic blood pressure (mmHg)	79.4 ± 8.6	78.1 ± 7.3	0.580
Body mass index (kg/m^2^)	23.4 ± 2.9	23.2 ± 3.7	0.787
Central corneal thickness (µm)	534.7 ± 36.3	540.3 ± 33.0	0.487
Axial length (mm)	24.9 ± 1.5	24.8 ± 1.4	0.867
Spherical equivalent (diopter)	−2.6 ± 3.6	−2.6 ± 3.2	0.950
Mean IOP (mmHg)	14.6 ± 2.5	14.7 ± 2.1	0.833
SD of IOP (mmHg)	2.2 ± 0.7	2.4 ± 0.7	0.396
Presence of disc hemorrhage (%)	16 (12.4%)	5 (15.2%)	0.771
Baseline MD of VF 24-2 (dB)	−3.9 ± 4.3	−4.5 ± 4.6	0.488
Baseline PSD of VF 24-2 (dB)	4.5 ± 3.9	5.8 ± 4.5	0.096
Follow-up duration (months)	97.1 ± 25.2	104.2 ± 24.1	0.148

IOP, intraocular pressure; MD, mean deviation; PSD, pattern standard deviation; SD, standard deviation; VF, visual field. Statistically significant differences between two groups (*p* < 0.05) by Student’s *t*-test for continuous variables or chi-squared test for categorical data are indicated in bold.

**Table 7:** Each retinal layer thickness according to the progression of visual field defects.

Findings Observed on OCT			Nonprogressors (*n* = 129)	Progressors (*n* = 33)	*p* Value
Microcystic macular changes			5 (3.9%)	7 (21.2%)	**0.003**
Epiretinal membrane			1 (0.8%)	4 (12.1%)	**0.006**
Each retinal layer thickness	Retina	Average (µm)	302.08 ± 15.32	303.88 ± 18.75	0.567
		Volume (mm^3^)	8.31 ± 0.43	8.33 ± 0.51	0.829
	RNFL	Average (µm)	24.00 ± 4.48	23.87 ± 4.35	0.879
		Volume (mm^3^)	0.81 ± 0.16	0.78 ± 0.14	0.478
	GCL	Average (µm)	34.91 ± 6.01	34.64 ± 6.29	0.827
		Volume (mm^3^)	0.95 ± 0.15	0.94 ± 0.16	0.783
	IPL	Average (µm)	30.57 ± 3.78	30.52 ± 4.39	0.943
		Volume (mm^3^)	0.82 ± 0.10	0.81 ± 0.11	0.839
	INL	Average (µm)	34.47 ± 2.55	35.72 ± 3.24	**0.019**
		Volume (mm^3^)	0.96 ± 0.06	0.99 ± 0.08	**0.015**

GCL, ganglion cell layer; INL, inner nuclear layer; IPL, inner plexiform layer; OCT, optical coherence tomography; RNFL, retinal nerve fiber layer. Statistically significant differences between two groups (*p* < 0.05) by Student’s *t*-test are indicated in bold.

**Table 8:** Multivariate analysis of factors associated with visual field progression.

Parameters	Trend-Based Analysis (MD Change/Year)				Event-Based Analysis (EMGT Criteria)			
	Univariate		Multivariate		Univariate		Multivariate	
	β Coefficient (95% CI)	*p* Value	β Coefficient (95% CI)	*p* Value	β Coefficient (95% CI)	*p* Value	β Coefficient (95% CI)	*p* Value
Presence of cold hand	−0.180 (−0.377~0.017)	0.073			2.681 (1.097~6.555)	**0.031**	2.283 (0.864~6.035)	0.096
Presence of microcystic macular changes	−0.349 (−0.631~−0.066)	**0.016**	−0.164 (−0.473~0.145)	0.297	6.677 (1.965~22.685)	**0.002**	4.037 (0.992~16.430)	0.051
Presence of ERM	−0.554 (−0.981~−0.127)	**0.011**	−0.286 (−0.755~0.183)	0.230	6.378 (1.902~163.874)	**0.012**	4.918 (0.419~57.681)	0.205
Average INL thickness	−0.043 (−0.070~−0.016)	**0.002**	−0.030 (−0.060~−0.001)	**0.045**	1.176 (1.018~1.357)	**0.027**	1.062 (0.893~1.262)	0.497

ERM, epiretinal membrane; INL, inner nuclear layer; MD, mean deviation. Multivariate regression analysis of factors associated with MD slope (dB/year) and multiple logistic regression analysis of factors associated with VF progression based on EMGT criteria were applied to the variables showing *p* value < 0.05 in each univariate analysis. The variables that were statistically significant in the multivariate regression analysis and multiple logistic regression analysis were indicated in bold.

**Table 9:** Factors associated with average inner nuclear thickness in patients with glaucoma.

Parameters	Total Patients				Patients without Microcystic Macular Changes			
	Univariate		Multivariate		Univariate		Multivariate	
	β Coefficient (95% CI)	*p* Value	β Coefficient (95% CI)	*p* Value	β Coefficient (95% CI)	*p* Value	β Coefficient (95% CI)	*p* Value
Age	0.029 (−0.002~0.061)	0.064			0.019 (−0.009~0.048)	0.186		
Sex	−0.460 (−1.312~0.392)	0.288			−0.621 (−1.387~0.145)	0.111		
Diabetes	0.841 (−0.334~2.016)	0.160			0.748 (−0.406~1.901)	0.202		
Systemic hypertension	−0.053 (−1.013~0.908)	0.914			0.070 (−0.802~0.942)	0.874		
Systolic BP (mmHg)	0.012 (−0.022~0.046)	0.489			0.009 (−0.024~0.043)	0.576		
Diastolic BP (mmHg)	−0.005 (−0.065~0.055)	0.869			−0.008 (−0.067~0.051)	0.787		
CCT (µm)	0.007 (−0.007~0.020)	0.336			−0.002(−0.014~0.010)	0.750		
Axial length (mm)	0.067 (−0.216~0.351)	0.639			−0.372 (−0.656~−0.088)	**0.011**	−0.310 (−0.583~0.037)	**0.026**
Mean IOP (mmHg)	0.072 (−0.105~0.249)	0.424			0.067 (−0.091~0.225)	0.401		
SD of IOP (mmHg)	1.081 (0.471~1.692)	**0.001**	0.861 (0.288~1.434)	**0.003**	0.815 (0.244~1.386)	**0.005**	1.107 (0.477~1.737)	**0.001**
Presence of optic disc hemorrhage	−0.735 (−2.029~0.559)	0.264			0.261 (−0.873~1.395)	0.650		
Baseline VF MD	−0.104 (−0.200~0.008)	**0.035**	0.049 (0.130~0.227)	0.593	−0.056 (−0.148~0.035)	0.225		
Baseline VF PSD	0.107 (0.002~0.211)	**0.046**	0.102 (0.090~0.294)	0.295	0.061 (−0.038~0.159)	0.226		
Presence of ERM	5.884 (3.591~8.176)	**<0.001**	4.331 (2.004~6.658)	**<0.001**	3.332 (0.012~6.652)	**0.049**	2.329 (−1.971~6.629)	0.285
Presence of microcystic macular changes	3.852 (2.336~5.368)	**<0.001**	2.315 (0.734~3.896)	**0.004**				

BP, blood pressure; CCT, central corneal thickness; ERM, epiretinal membrane; IOP, intraocular pressure; MD, mean deviation; PSD, pattern standard deviation; SD, standard deviation; VF, visual field. The variables that were statistically significant in the univariate and multivariate regression analysis were indicated in bold.

**Table 10:** Logistic regression analysis of factors associated with the presence of microcystic macular changes.

Variables	Univariate		Multivariate	
	β Coefficient (95% CI)	*p* Value	β Coefficient (95% CI)	*p* Value
Presence of diabetes	6.895 (2.016~23.580)	**0.002**	5.838 (1.301~26.201)	**0.021**
Presence of ERM	24.667 (3.647~1666.834)	**0.001**	14.590 (0.364~156.115)	**0.027**
Baseline MD	0.860 (0.773~0.957)	**0.006**	0.906 (0.796~1.041)	0.169
Higher IOP fluctuation	12.571 (1.583~99.835)	**0.017**	11.089 (1.288~95.430)	**0.028**

ERM, epiretinal membrane; IOP, intraocular pressure; MD, mean deviation. The variables that were statistically significant in the logistic regression analysis are indicated in bold.

## Data Availability

The data presented in this study are available on request from the corresponding author. The data are not publicly available due to privacy.
